# Region-specific heterogeneity in neuronal nuclear morphology in young, aged and in Alzheimer’s disease mouse brains

**DOI:** 10.3389/fcell.2023.1032504

**Published:** 2023-02-01

**Authors:** Soumen Das, Narendrakumar Ramanan

**Affiliations:** Centre for Neuroscience, Indian Institute of Science, Bangalore, India

**Keywords:** nuclear morphology, nuclear size, neuronal nucleus, aging, Alzheimer’s disease

## Abstract

Neurons in the mammalian brain exhibit enormous structural and functional diversity across different brain regions. Compared to our understanding of the morphological diversity of neurons, very little is known about the heterogeneity of neuronal nuclear morphology and how nuclear size changes in aging and diseased brains. Here, we report that the neuronal cell nucleus displays differences in area, perimeter, and circularity across different anatomical regions in the mouse brain. The pyramidal neurons of the hippocampal CA3 region exhibited the largest area whereas the striatal neuronal nuclei were the smallest. These nuclear size parameters also exhibited dichotomous changes with age across brain regions–while the neocortical and striatal neurons showed a decrease in nuclear area and perimeter, the CA3 neurons showed an increase with age. The nucleus of parvalbumin- and calbindin-positive interneurons had comparable morphological features but exhibited differences between brain regions. In the context of activity-dependent transcription in response to a novel environment, there was a decrease in nuclear size and circularity in c-Fos expressing neurons in the somatosensory cortex and hippocampal CA1 and CA3. In an APP/PS1 mutant mouse model of Alzheimer’s disease (AD), the neuronal nuclear morphology varies with plaque size and with increasing distance from the plaque. The neuronal nuclear morphology in the immediate vicinity of the plaque was independent of the plaque size and the morphology tends to change away from the plaque. These changes in the neuronal nuclear size and shape at different ages and in AD may be attributed to changes in transcriptional activity. This study provides a detailed report on the differences that exist between neurons in nuclear morphology and can serve as a basis for future studies.

## Introduction

There are approximately 75 million neurons in the mouse brain and these exhibit diverse heterogeneity in both structure and function across different regions (Herculano-Houzel et al., 2006; Ero et al., 2018). Different structural characteristics bestow neurons with different functionalities and connectivity within the brain. Neurons exhibit remarkable ability to undergo morphological alterations in response to environmental changes and this is governed by distinct patterns of gene expression. These morphological changes that include dendritic structure and synaptic connections govern the ability of neurons to fine-tune their connectivity and regulate information processing (Muller et al., 2002). The chromatin architecture in the nucleus plays an important role in regulating gene expression critical for neuronal structure and function. In addition to the dynamic nature of chromatin, the neuronal nucleus itself has been shown to undergo changes in its morphology during neuronal differentiation and development (Smith et al., 2011; Ankam et al., 2018; Roubinet et al., 2021). In contrast to the wealth of information on the neuronal soma size, axonal length, dendritic complexity, spine type and density and electrophysiology, very little is known about nuclear morphological changes among different neuronal populations in the brain.

Previous studies have observed differences in the morphology of neuronal nuclei, both in humans and in rodents, across different brain regions in various pathological conditions (Rajkowska et al., 1998; Edens et al., 2013; Ito and Takizawa, 2018; Ishunina et al., 2019; Alcala-Vida et al., 2021). In humans, there exists a region-specific heterogeneity in neuronal nuclear volume. Neurons in the putamen have the smallest nuclear volume whereas the neurons in the substantia nigra have the largest nuclear volume (Wegiel et al., 2015). In 4-8-year-old autistic children, several brain regions displayed deficits in nuclear volume whereas in older autistic individuals, there were relatively fewer or no regions with significant changes in the neuronal nuclei volume (Wegiel et al., 2015). In brains from vascular dementia and Alzheimer’s disease (AD) patients, the neuronal nuclei size was significantly larger in the hypothalamus when compared to brains of control healthy individuals (Ishunina et al., 2019). Another study on *post-mortem* brain samples from AD, Lewy body dementia (LBD) and control patients reported a significant reduction in oligodendrocyte nuclei diameter while no change in mean neuronal nuclei diameter was observed in the hippocampi of AD and LBD brains when compared to control samples (Gagyi et al., 2012).

In contrast to the observations made in human *post-mortem* brain samples, much less is known about the nuclear morphology in model systems including rodents. Previous observations in male Wistar rats showed that the diameter of neocortical neuronal nuclei increases when the animals were reared in an enriched environment compared to a sensory deprived environment (Diamond et al., 1975). However, the hippocampal neuronal nuclei size did not show any significant change in response to environmental stimulation (Walsh and Cummins, 1979). Likewise, three-dimensional analysis of rat hippocampal neurons revealed various degrees of infoldings of the nucleus depending on the nature of the extracellular stimuli (Wittmann et al., 2009). Despite these findings, very little is known about the region-specific differences in nuclear morphology in neurons. In this study, we sought to analyze nuclear morphology of excitatory and inhibitory neurons in different brain regions to determine whether the neuronal soma morphology, aging, and stimulus-induced neuronal activity influences nuclear morphology in neurons. We found differences in the area, perimeter, and circularity among both excitatory and inhibitory neurons in different brain regions that varied with age. Stimulus-dependent neuronal activation in response to a novel environment caused a decrease in nuclear size and a change in shape. In addition, in a mouse model of Alzheimer’s disease (AD), we found that the β-amyloid plaques only affected nuclear morphology of neurons that were closer to the plaques.

## Materials and methods

### Animals

C57BL/6J (Strain #000664) and APPSwe/PSen1dE9 (APP/PS1) (MMRRC Strain #034829-JAX) mice were used for all experiments and maintained by internal breeding in individually ventilated cages (Techniplast, Italy) under conventional conditions (23°C ± 2°C, relative humidity 50 ± 10%, 12-h light/dark cycle) and had *ad libitum* access to food and water. All the procedures in this study were performed according to the rules and guidelines of the Committee for the Purpose of Control and Supervision of Experimental Animals (CPCSEA), India. The research protocol was approved by the Institutional Animal Ethics Committee (IAEC) of the Indian Institute of Science. Genotyping was performed by PCR analysis using tail-tip DNA.

### Novel cage exploration

Animals were habituated in their home cage in the behavior room for at least 3 days. After three-four days, animals were placed in a novel cage equipped with multiple novel objects for the animals to explore for 60 min, following which, the animals were immediately perfused, and brains were isolated for analysis. The control animals stayed in the home cage during this time.

### Immunohistochemistry

The mice were anesthetized with i.p. injection of Avertin (2,2,2-tribromoethanol, Cat. No. T48402, and 2-methyl-2-butanol, Cat. No. 240486, Sigma Aldrich, MO, United States) and perfused transcardially with ice-cold 4% paraformaldehyde (PFA) in phosphate buffered saline (PBS, pH 7.4). Brains were removed and post-fixed for 12–16 h in 4% PFA in PBS at 4°C. The brains were then cryoprotected in 30% sucrose prepared in 0.1M PBS for 2–3 days at 4°C. The brains were snap frozen using isopentane and stored at −80°C until use. 35–40 µm thick cryosections were cut for immunostaining. In these sections some nuclei were likely still intact while others could be cut at different nuclear heights. The frequency of intact nuclei found within a section for cell types with small nuclei should be higher compared to cell types with large nuclei. The sections were washed in PBS, incubated in blocking solution (3% bovine serum albumin, 0.3% Triton X-100 and 1% normal horse serum) in 0.1M PBS at room temperature. Sections were then incubated overnight in the primary antibody at 4°C. The sections were washed in PBS (6 × 10 min) and incubated in appropriate secondary antibodies for 1 h at room temperature. Sections were then washed again in PBS (4 × 10 min) and mounted with mounting media containing DAPI (Cat. No. H-1200, Vector Laboratories, CA, United States). The following antibodies were used: mouse anti-LMN1 (1:100, DSHB, #LMN1), rabbit anti-Lamin A/C (1:100, Abclonal, #A0249), mouse anti-NeuN (1:500, Millipore, #MAB-377), chicken anti-NeuN (1:500, Synaptic systems, #266006), mouse anti-MOAB-2 (1:500, Novus Bio, #NBP2-13075) and guinea pig anti-c-Fos (1:500, Synaptic systems, #226308). Secondary antibodies include Alexa 488 or Alexa 594 conjugated goat/donkey anti-rabbit antibody, Alexa 488, Alexa 594 or Alexa 647 conjugated goat/donkey anti-mouse antibody and Alexa 488 conjugated goat/donkey anti-chicken antibody (All secondary antibodies were obtained from Jackson ImmunoResearch and Invitrogen). Anti-Lmn A/C and Anti-Lmn-1 gave very similar staining and Lmn A/C, or Lmn-1 were used inter-changeably depending on the host compatibility of the antibodies used in co-labeling. Similar numbers of nuclei were observed within the region of interest (ROI) that were considered for analyses.

### Image acquisition

Fluorescent images (×2, ×20 or ×40 magnification) were captured using a Nikon Eclipse 80i epifluorescence microscope with photometrics CoolSNAP EZ camera and Metamorph software (Molecular Devices). All the image panels and figures were prepared using ImageJ and Adobe illustrator.

### Quantification and statistical analysis

All the quantifications (area, perimeter, circularity, and cell counts) were performed using ImageJ. All the images were scaled based on the objective magnifications. Measurements were set for area, perimeter, and shape descriptors from the analyze menu in ImageJ. Polygon selection from the tools bar was used to draw around the nuclear envelope (based on Lamin staining) and measure the nuclear area, perimeter, and circularity. A total of 80–150 nuclei for each brain region were used for measurements (*n* = 3 mice). For AD sample quantification, four concentric circles (at an interval of 20 µm) were drawn around the plaques of specific radii and the nuclear area, perimeter and circularity were determined for each circle. For quantification, multiple plaques in close proximity were avoided and only isolated plaques were considered. All the statistics and graphs were generated using GraphPad Prism 8. Data represented in graphs as mean ± SEM. Each data point in the graphs represents an individual nucleus unless mentioned otherwise. Sample sizes and other statistical details are defined in the figure legends.

## Results

### Neuronal nuclear morphology in the neocortex and striatum in young and aged animals

**FIGURE 1:**
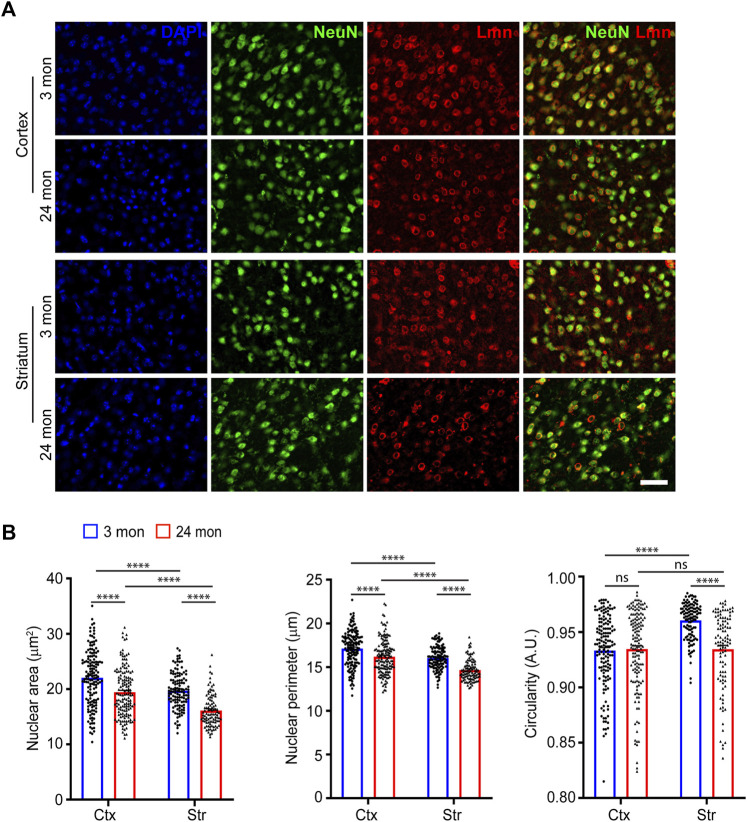
Neuronal nuclear morphology in the neocortex and striatum. **(A)** Immunostaining of brain sections from 3-month and 24-month-old mice using anti-NeuN (green), anti-Lmn (red) along with DAPI (blue) (Scale bar, 20 µm). **(B)** Mean neuronal nuclear area, perimeter, and circularity in the neocortex (Ctx) and striatum (Str). One-Way ANOVA (*p < 0.05, **p < 0.01, ***p < 0.001 and ****p < 0.0001). *n* = 3 mice. Data represented as mean ± SEM.

During development, neural stem cells generate neurons by asymmetric cell division and the newborn neurons have shown to exhibit changes in nuclear morphology (Ankam et al., 2018; Roubinet et al., 2021). We first asked whether neurons in the neocortex and striatum in the adult brain differed in their sizes or have a uniform morphology. For this, we immunostained coronal brain sections from 3-month-old mice for the neuronal marker, NeuN and the nuclear membrane marker, Lamin (Figure 1A). We measured the nuclear area, perimeter, and circularity from the neurons in the neocortex and striatum. We found that the shape and size of the nuclei in these neurons were not uniform and exhibited differences between these regions. The neocortical neuronal nuclei were larger than those of striatal neurons (Figure 1B; Supplementary Figure S1; Table 1). When circularity was considered, the nuclei of striatal neurons were the most circular while the neocortical neurons had less circular nuclei (Figure 1B; Supplementary Figure S1; Table 1).

**TABLE 1:** Nuclear area, perimeter, and circularity of neurons in 3-month and 24-month mice.

	Cortex (150 cells)	Striatum (120 cells)	CA1 (80 cells)	CA3 (80 cells)
Nuclear area (3 months)	22.03 ± 0.413	19.71 ± 0.293	20.36 ± 0.362	23.2 ± 0.355
Nuclear perimeter (3 months)	17.11 ± 0.166	16.05 ± 0.12	16.53 ± 0.134	17.44 ± 0.132
Circularity (3 months)	0.933 ± 0.003	0.961 ± 0.002	0.938 ± 0.003	0.957 ± 0.002

	Cortex (150 cells)	Striatum (100 cells)	CA1 (120 cells)	CA3 (90 cells)
Nuclear area (24 months)	19.42 ± 0.361	16.11 ± 0.293	21.16 ± 0.392	26.23 ± 0.596
Nuclear perimeter (24 months)	16.19 ± 0.166	14.66 ± 0.123	16.86 ± 0.154	18.5 ± 0.210
Circularity (24 months)	0.935 ± 0.003	0.934 ± 0.003	0.929 ± 0.003	0.957 ± 0.002

*n* = 3 mice; Data shown as Mean ± SEM.

Previous studies have shown that cellular senescence affects nuclear morphology (Pathak et al., 2021; Heckenbach et al., 2022). We therefore sought to characterize nuclear morphology during aging. For this, brain sections from aged (24 months) mice were immunostained for NeuN and Lamin and different morphological features of the nucleus were compared (Figure 1A). On comparing nuclear size and circularity of neurons at 3 months of age with those at 24 months, we found that the nuclear size for neocortical and striatal neurons was reduced at 24 months of age compared to young neurons (Figure 1B; Table 1). We also observed a significant reduction in nuclear circularity for the striatal neurons with age (Figure 1B).

### Neuronal nuclear morphology in the hippocampus in young and aged animals

**FIGURE 2:**
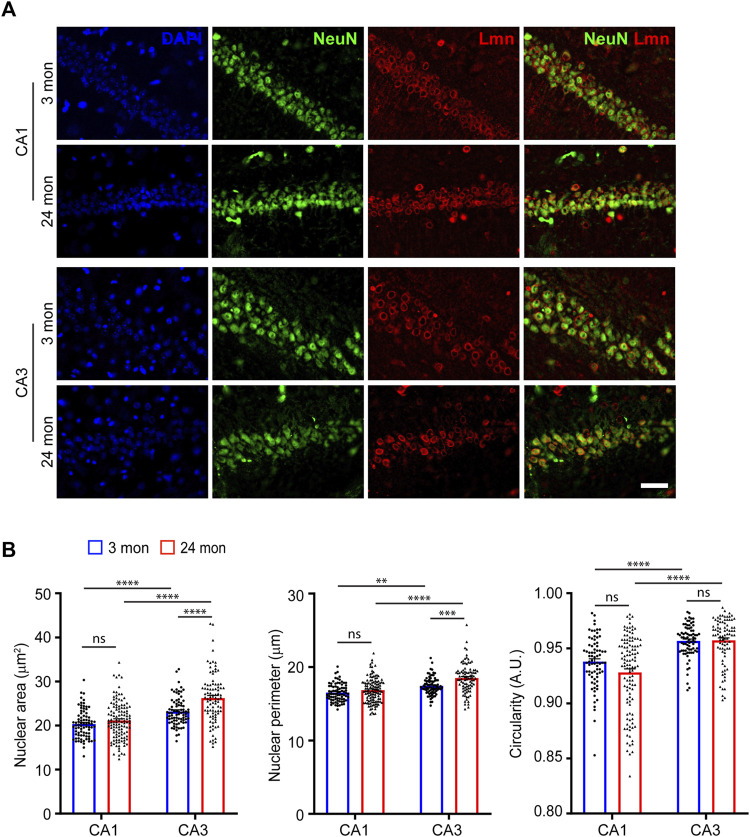
Neuronal nuclear morphology of hippocampal neurons. **(A)** Immunostaining of brain sections from 3-month and 24-month-old mice using anti-NeuN (green), anti-Lmn (red) along with DAPI (blue) (Scale bar, 20 µm). **(B)** Mean neuronal nuclear area, perimeter, and circularity in the neocortex, hippocampus (CA1 & CA3) and striatum. One-Way ANOVA (*p < 0.05, **p < 0.01, ***p < 0.001 and ****p < 0.0001). *n* = 3 mice. Data represented as mean ± SEM.

We next investigated the nuclear morphology of hippocampal CA1 and CA3 pyramidal neurons (Figure 2A). Immunostaining for Lamin revealed that the hippocampal CA3 pyramidal neurons exhibited the largest nuclear size at 3 months of age (Figure 2B). We found that even at 24-months of age, the CA3 pyramidal neurons had the largest nuclear area (Figure 2B, Supplementary Figure S2; Table 1). However, unlike at 3 months of age, the CA3 pyramidal neurons at 24-months were the most circular while the CA1 pyramidal neurons were the least circular (Figure 2B, Supplementary Figure S2; Table 1). In contrast, the CA3 neuronal nuclei showed an increase in size as the animal ages (Figure 2B, Supplementary Figure S2; Table 1). When circularity was measured, there was no change for hippocampal CA1 and CA3 neurons between the two age groups (Figure 2B; Supplementary Figure S2). Together, the above observations suggested that the morphology of neocortical and striatal neuronal nuclei were the most affected by age when compared to hippocampal neurons. These findings also suggest that aging neurons vary widely in their nuclear morphology, and this could likely be attributed to their overall cellular shape, architecture and physiology (Chen et al., 2015).

### Neuronal nuclear morphology in interneurons

**FIGURE 3:**
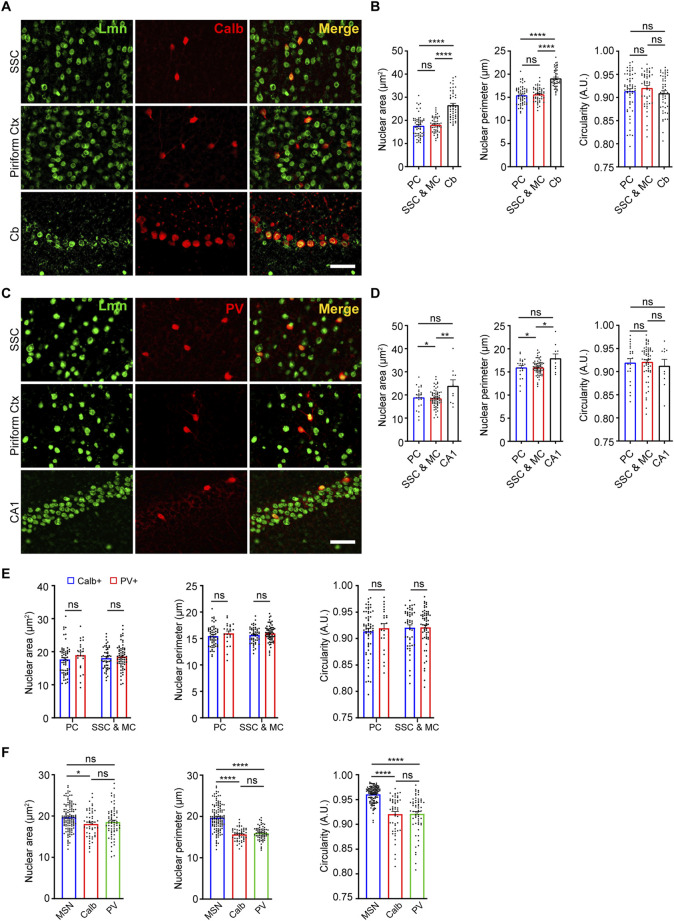
Nuclear morphology of Calbindin-positive and Parvalbumin-positive neurons. **(A)** Immunostaining of brain sections from 3-month-old mice using anti-Lmn (green), anti-Calbindin (red) along with DAPI (blue). Scale bar, 25 µm. **(B)** Mean nuclear area, nuclear perimeter, and nuclear circularity of Calbindin-positive neurons in the somatosensory cortex, motor cortex, piriform cortex, and cerebellum. **(C)** Immunostaining of brain sections from 3-month-old mice using anti-Lmn (green), anti-Parvalbumin (red) along with DAPI (blue). Scale bar, 25 µm. **(D)** Mean neuronal nuclear area, perimeter, and circularity of Parvalbumin-positive neurons in the somatosensory cortex, motor cortex, piriform cortex and hippocampal CA1. **(E)** Comparison of mean nuclear area, perimeter, and circularity between calbindin-positive and parvalbumin-positive interneurons in the piriform cortex, somatosensory cortex, and motor cortex at 3 months. **(F)** Comparison of mean nuclear area, perimeter, and circularity of striatal medial spiny neurons, Calbindin- and Parvalbumin-positive interneurons in the somatosensory, and motor cortex at 3 months of age. One-Way ANOVA (*p < 0.05, **p < 0.01, ***p < 0.001 and ****p < 0.0001). *n* = 3 mice. Data represented as mean ± SEM. PC, piriform cortex; SSC, somatosensory cortex; MC, motor cortex; MSN, medial spiny neurons; Calb, calbindin; PV, parvalbumin.

We next sought to determine the nuclear morphology of two different populations of interneurons in the brain and how it is influenced by age. First, we determined the nuclear area, perimeter, and circularity for Calbindin-positive interneurons. For this, we immunostained coronal brain sections using antibodies against Calbindin and Lamin at 3 months of age and measured the morphological parameters based on lamin staining (Figure 3A). When size was compared, the cerebellar Purkinje neurons displayed the largest nuclear area and perimeter among the Calbindin-positive interneurons in the brain (Figure 3B, Supplementary Figure S3; Table 2). However, there was no significant difference in nuclear circularity among the Calbindin-positive interneurons in different neocortical regions and cerebellum (Figure 3B). Next, we immunostained 3-month-old brain sections using antibodies against another interneuron marker, Parvalbumin and Lamin (Figure 3C). We found that the Parvalbumin-positive interneurons in the hippocampal CA1 region exhibited the largest nucleus than those in the neocortex (Figure 3D, Supplementary Figure S3; Table 2). We next compared the nuclear morphology between the Calbindin-positive and Parvalbumin-positive interneurons in the neocortex. We found no discernible differences in all the parameters of morphology compared, suggesting that there is more uniformity in nuclear morphology among these two classes of interneurons in the neocortex (Figure 3E; Table 2). However, the GABAergic medial spiny neurons in the striatum displayed a larger size and more circular nuclei, when compared to the Calbindin-positive and Parvalbumin-positive interneurons in the neocortex (Figure 3F; Table 2).

**TABLE 2:** Nuclear area, perimeter, and circularity of calbindin-positive and parvalbumin-positive neurons.

Calbindin-positive neurons	Piriform cortex (60 cells)	SSC & MC (50 cells)	Cerebellum (50 cells)
Nuclear area	17.67 ± 0.592	18.09 ± 0.471	26.59 ± 0.823
Nuclear perimeter	15.46 ± 0.249	15.66 ± 0.216	19.11 ± 0.280
Circularity	0.914 ± 0.006	0.921 ± 0.005	0.909 ± 0.006

Parvalbumin–positive neurons	Piriform cortex (22 cells)	SSC & MC (66 cells)	CA1 (10 cells)
Nuclear area	18.98 ± 1.005	18.55 ± 0.459	23.99 ± 2.51
Nuclear perimeter	15.98 ± 0.448	15.97 ± 0.196	17.96 ± 0.927
Circularity	0.919 ± 0.009	0.921 ± 0.005	0.913 ± 0.014

*n* = 3 mice; Data shown as Mean ± SEM.

### Nuclear size and shape vary following novel environment exploration

**FIGURE 4:**
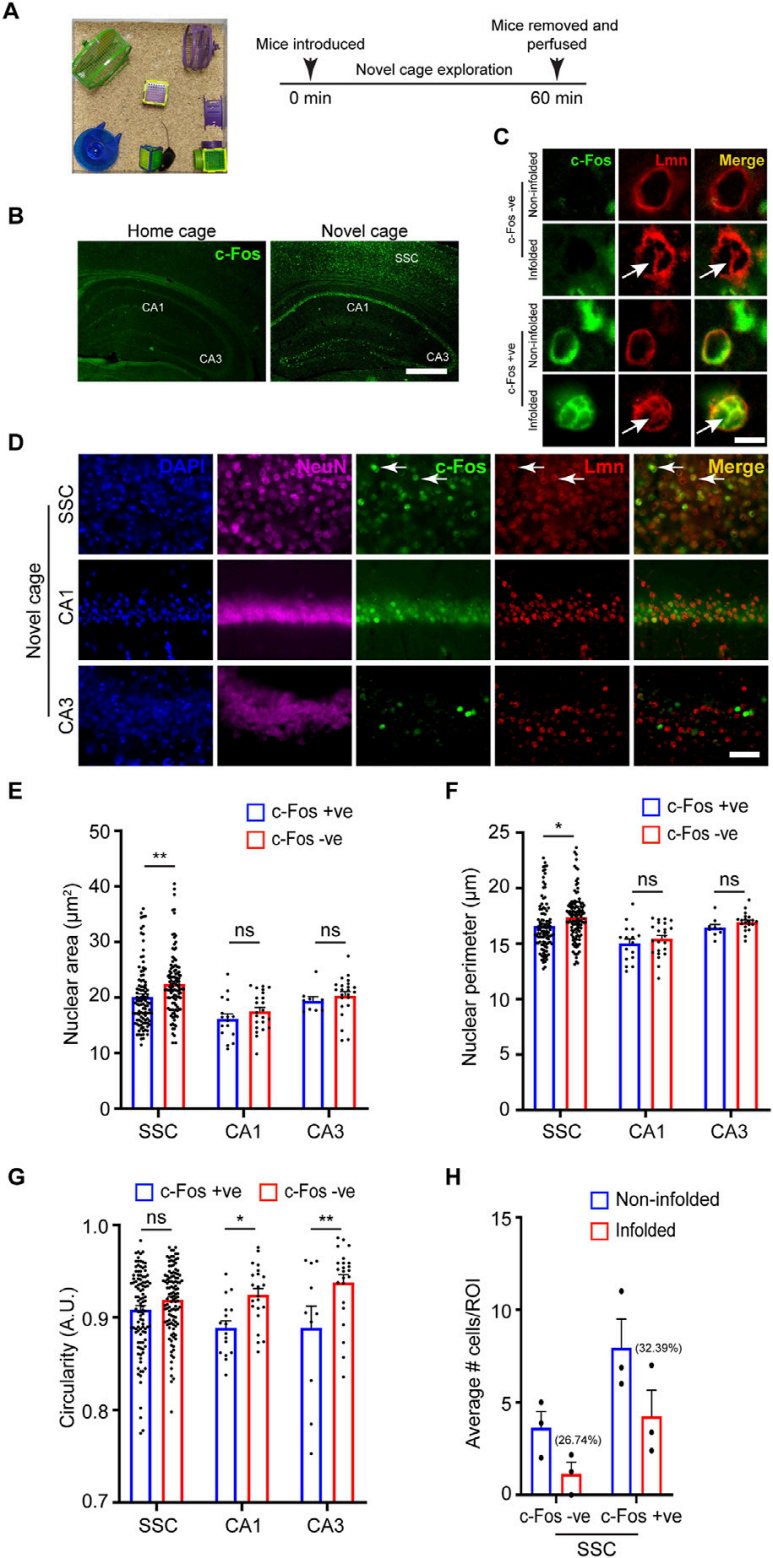
Neuronal nuclear morphology following novel environment exploration. **(A)** Schematic representation of the novel environment and the experimental paradigm. **(B)** Immunostaining for the activity-induced immediate early gene c-Fos (green) shows robust upregulation of c-Fos in the neurons in the somatosensory cortex and hippocampal regions (white arrows) in novelty explored mice compared to home-caged control mice (Scale bar, 500 µm). **(C)** Immunostaining using anti-c-Fos (green) and anti-Lmn (red) shows the presence of nuclear infoldings in neurons of the somatosensory cortex (white arrows) (Scale bar, 5 µm). **(D)** Immunostaining using anti-NeuN (magenta), anti-cFos (green) and anti-Lmn (red) with DNA staining using DAPI (blue). Scale bar, 25 µm. White arrows indicate c-Fos-positive neurons. **(E–G)** Comparison of mean nuclear area **(E)**, perimeter **(F)**, and circularity **(G)** of c-Fos-negative and c-Fos-positive neurons in the somatosensory cortex and hippocampal CA1 and CA3 regions. **(H)** Comparison of average number of non-infolded vs. infolded nuclei in c-Fos-positive and c-Fos-negative cells. ROI is 0.01 mm^2^, *n* = 3 mice. Two-Way ANOVA (*p < 0.05, **p < 0.01, ***p < 0.001 and ****p < 0.0001). Data represented as mean ± SEM. S. cortex, somatosensory cortex.

Neuronal activity is a major regulator of neuronal circuit formation and long-term adaptive responses in the nervous system (Zhang and Poo, 2001; Hua and Smith, 2004). Neuronal activity has been shown to regulate nuclear architecture and chromatin accessibility in the brain (Takizawa and Meshorer, 2008; Su et al., 2017) and genome architecture can regulate gene expression in several ways (Brookes and Riccio, 2019). Previous studies have shown that synaptic activity in cultured neurons and organotypic slices induced nuclear membrane in-folding and chromatin reorganization (Wittmann et al., 2009; Peter et al., 2021). We asked whether neuronal activity influences nuclear morphology *in vivo*. Novel environment exploration has been shown to promote immediate early gene expression and structural changes critical for learning and memory (Handa et al., 1993; Papa et al., 1993). To induce novelty-induced gene expression, we allowed 6-8-month-old mice to explore a novel enriched environment (NEE) for 60 min (Figure 4A). The neurons activated in this paradigm were identified by the expression of the immediate-early gene, c-Fos, which has been shown to be rapidly induced in activated neurons (Handa et al., 1993; Papa et al., 1993). We immunostained coronal brain sections for NeuN, cFos and Lamin (Figures 4B–D). We found robust activation of c-Fos protein in a vast population of neurons in the somatosensory cortex and place cells in the hippocampal CA1 region in the NEE mice compared to home-caged mice consistent with previous observations (Figure 4B) (Papa et al., 1993). We next investigated the potential changes in nuclear morphology in c-Fos-negative *versus* the c-Fos-positive neurons in the somatosensory cortex. We found that the nucleus area and perimeter were smaller in the c-Fos-positive neurons compared to c-Fos-negative neurons (Figures 4E, F; Table 3). However, there was no change in the circularity of the nucleus in the neurons in the somatosensory cortex while the c-Fos-positive CA1 and CA3 neurons showed a decrease in circularity compared to c-Fos-negative cells (Figure 4G; Table 3). An earlier study had shown that synaptic activity caused complex infoldings in hippocampal neurons (Wittmann et al., 2009). We therefore looked for similar infolded structures in the nucleus of c-Fos-positive *versus* c-Fos-negative neurons in the somatosensory cortex (Figure 4C). We found many c-Fos-negative cells (26.74%) with an infolded nucleus. However, this increased to 32.39% in c-Fos-positive neurons (Figure 4H). Together these observations indicate that neuronal activity in response to external stimuli induces complex changes to nuclear morphology, which could play an important role in the regulation of gene expression.

**TABLE 3:** Nuclear area, perimeter, and circularity of c-Fos-positive and c-Fos-negative neurons in somatosensory (SSC) and hippocampal CA1 and CA3 regions.

Somatosensory cortex (SSC)	c-Fos positive (105 cells)	c-Fos negative (114 cells)
Nuclear area	20.07 ± 0.558	22.42 ± 0.519
Nuclear perimeter	16.58 ± 0.225	17.39 ± 0.197
Circularity	0.909 ± 0.004	0.919 ± 0.003

Hippocampal CA1 neurons	c-Fos positive (16 cells)	c-Fos negative (23 cells)
Nuclear area	16.14 ± 0.932	17.54 ± 0.708
Nuclear perimeter	15.01 ± 0.416	15.43 ± 0.312
Circularity	0.889 ± 0.008	0.925 ± 0.007

Hippocampal CA3 neurons	c-Fos positive (10 cells)	c-Fos negative (23 cells)
Nuclear area	19.38 ± 0.752	20.31 ± 0.754
Nuclear perimeter	16.44 ± 0.282	16.96 ± 0.184
Circularity	0.889 ± 0.024	0.938 ± 0.008

*n* = 3 mice. Data shown as Mean ± SEM.

### Neuronal nuclear morphology around plaques in Alzheimer’s disease

**FIGURE 5:**
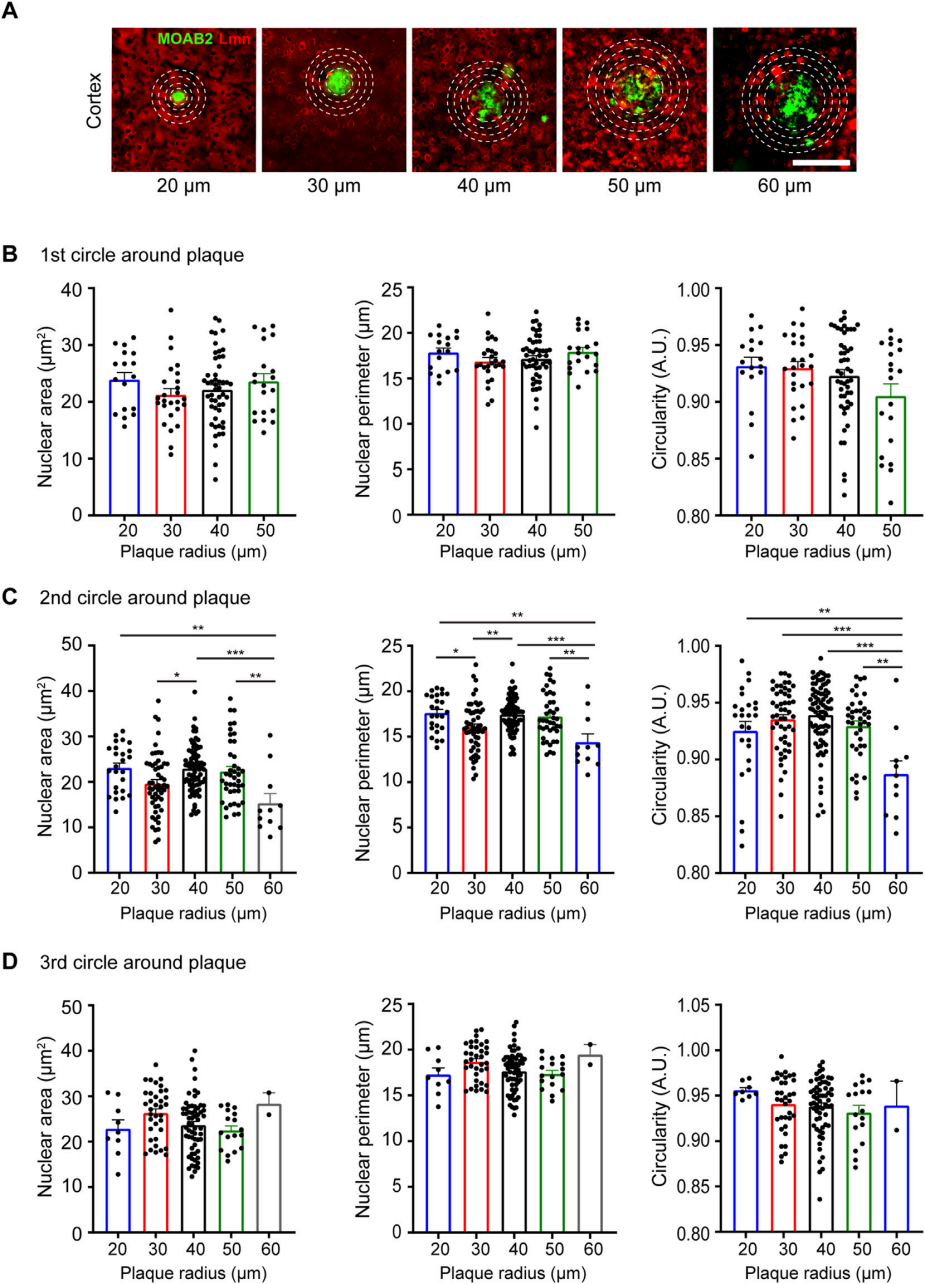
Neuronal nuclear morphology around plaques in the APP/PS1 transgenic mice at 6 months of age. **(A)** Immunostaining using anti-NeuN (blue), anti-MOAB2 (green; stains plaques) and anti-Lmn (red). Scale bar, 30 µm. **(B–D)** Mean nuclear area, perimeter, and circularity at 20 µm radius **(B)**, 40 µm radius **(C)** and 60 µm radius **(D)** from plaques of different sizes. Neuronal nuclei morphology characterized for the following plaque radii: 20, 30, 40, 50, and 60 μm *n* = 3 mice. One-Way ANOVA (*p < 0.05, **p < 0.01, ***p < 0.001 and ****p < 0.0001). Data represented as mean ± SEM.

Neurodegenerative disorders have profound effects on brain architecture that include neuronal cell loss and gross structural changes in various cell types (Dugger and Dickson, 2017). Neurons in the setting of neurodegeneration exhibit alterations in dendritic branching, spine structure and density and synapse numbers (Blanpied and Ehlers, 2004; Knobloch and Mansuy, 2008). Together these changes affect activity-dependent gene transcription critical for synaptic plasticity and cognition. Previous studies have observed changes in nuclear morphology in neurons in the hypothalamus and in specific neuronal populations in the hippocampus in human *post-mortem* brains (Gagyi et al., 2012; Ishunina et al., 2019). However, whether the extracellular plaques influence nuclear morphology is not known. To study this, we immunostained 6-month-old coronal brain sections from the APP/PS1 mutant mice using the β-amyloid antibody, anti-MOAB2 to identify extracellular plaques, along with NeuN and Lamin (Youmans et al., 2012) (Figure 5A). We then analyzed the nuclear morphology of the neocortical neurons at increasing distances from the plaques of different sizes. Four concentric circles of specific radii were drawn around each plaque and the nuclear size and circularity were estimated at an interval of 20 µm from the previous circle (Figure 5A). We observed that the neuronal nuclear morphology in the immediate vicinity (20 µm distance) of the plaque was independent of the size of the plaque (Figure 5B). However, all the three parameters of nuclear morphology that were measured showed changes as one moved away from the plaque with the maximum change seen for plaques of the largest size considered (60 µm radius) (Figure 5C). Taken together, the maximum change in morphological parameters was observed for the neurons located in the second circle (40 µm distance) independent of the plaque size whereas there was no significant change observed for the first (20 µm distance) and last circle (60 µm distance) around the plaque (Figures 5B–D; Table 4).

**TABLE 4:** Nuclear area, perimeter, and circularity of neurons at specific distance from plaques of varying sizes.

1st circle	20 µm (17 cells)	30 µm (26 cells)	40 µm (50 cells)	50 µm (20 cells)	60 µm
Nuclear area	23.91 ± 1.28	21.24 ± 1.11	22.12 ± 0.86	23.6 ± 1.39	—
Nuclear perimeter	17.86 ± 0.49	16.86 ± 0.44	17.16 ± 0.36	17.94 ± 0.47	—
Circularity	0.932 ± 0.01	0.930 ± 0.01	0.923 ± 0.01	0.905 ± 0.01	—

2nd Circle	20 µm (25 cells)	30 µm (54 cells)	40 µm (82 cells)	50 µm (41 cells)	60 µm (11 cells)
Nuclear area	23.11 ± 1.04	19.62 ± 0.91	22.95 ± 0.56	22.32 ± 1.08	15.32 ± 2.11
Nuclear perimeter	17.61 ± 0.41	16.05 ± 0.36	17.45 ± 0.21	17.23 ± 0.40	14.41 ± 0.91
Circularity	0.925 ± 0.01	0.936 ± 0.004	0.939 ± 0.003	0.930 ± 0.004	0.887 ± 0.01

3rd Circle	20 µm (9 cells)	30 µm (36 cells)	40 µm (61 cells)	50 µm (17 cells)	60 µm (2 cells)
Nuclear area	22.84 ± 1.97	26.31 ± 0.93	23.64 ± 0.78	22.47 ± 0.99	28.35 ± 2.40
Nuclear perimeter	17.28 ± 0.73	18.67 ± 0.34	17.64 ± 0.29	17.35 ± 0.39	19.48 ± 1.10
Circularity	0.96 ± 0.003	0.94 ± 0.005	0.94 ± 0.004	0.931 ± 0.01	0.94 ± 0.03

*n* = 3 mice. Data shown as Mean ± SEM.

## Discussion

Neurons in the mammalian brain exhibit enormous diversity in their morphology and physiology. Over the years, studies have reported differences in soma size and structure, dendritic complexity, spine type and density, and axonal length. However, information about neuronal nuclear morphology in the adult brain and how it may be influenced by aging and in the setting of neurodegenerative diseases is limited. In this study, we found that the neurons in different regions in the adult brain exhibited differences in size and circularity with CA3 pyramidal neurons in the hippocampus having a larger nucleus. With aging, the nucleus size showed a decrease in neurons in hippocampal CA1, neocortex and striatum while the CA3 neuronal nucleus showed an increase. In contrast, the calbindin and parvalbumin interneurons showed differences in nuclear morphology across different brain regions. Neuronal activity-induced by exposure to a novel environment resulted in a decrease in nuclear area but caused an increase in the number of nuclei with infoldings. In a mouse model of Alzheimer’s disease, the amyloid plaques influenced the nuclear area only at a specific distance.

Genome architecture is critical for regulating gene expression that is essential for the development and function of the nervous system (Ito and Takizawa, 2018; Brookes and Riccio, 2019). Critical insights into genome architecture and changes to the chromatin landscape have come from studies that used confocal 3D reconstructions, super-resolution microscopy and electron microscopy techniques to reveal finer details of the sub-nuclear structures (Solovei et al., 2004; Grabowska et al., 2022; Rose et al., 2022). During differentiation and development, the dynamic changes in the genome structure have a strong effect on the shape and geometry of the nucleus (Santama et al., 1996; Brookes and Riccio, 2019). Previous studies have shown that cell morphology could influence nuclear shape and size and nuclear shape could have potential impacts on cellular functions (Huber and Gerace, 2007; Chen et al., 2015; Skinner and Johnson, 2017). The pyramidal neurons are among the largest diameter neurons in the mammalian cortex. We found differences in the size and circularity among these neurons in the neocortex and hippocampus with the CA3 pyramidal neurons showing the largest area and this could be differences in the size of their respective perikaryons. Comparison with the GABAergic medial spiny neurons, the major neuronal population in the striatum, showed that the striatal neurons were the smallest but most circular. These differences in the nuclear size between pyramidal neurons and striatal neurons could be attributed to the differences in the sizes of their respective soma (Meitzen et al., 2011; Bicanic et al., 2017; Gilman et al., 2017).

Aging has a strong influence on neuronal morphology and can cause a decrease in the nucleus-cytoplasmic ratio, shrinkage of dendritic arbor and loss of dendritic spines (Peinado et al., 1993; Ledda et al., 2000; Dickstein et al., 2007; Pannese, 2011). Recent studies have shown that both the genome and neuronal nuclear architectures are affected in the aging brain (Schlachetzki et al., 2020; Pathak et al., 2021; Heckenbach et al., 2022) and the nuclear morphological changes can be used as a deep learning predictor of cellular senescence (Heckenbach et al., 2022). We found that the nuclear size was decreased in the neocortical and striatal neurons at 24-months compared to 3-months of age, whereas the CA3 neurons showed an increase. Our findings suggest that alterations in nuclear morphology could indeed serve as a great tool for identifying senescent neurons, but these changes may not be uniform across neuronal populations and may vary regionally.

Neurons constantly respond to changes in their environment by activating specific patterns of gene expression and this is critical for experience-dependent behavioral changes. Gene expression in neurons has been shown to be regulated by both changes in chromatin conformation and movement of the gene loci (Brookes and Riccio, 2019; Peter et al., 2021). Earlier studies in rats on the influence of activity-dependent transcription, in response to a novel enriched environment, have made different observations (Diamond et al., 1967; Diamond et al., 1975; Walsh and Cummins, 1979). The cortical neurons in the visual cortex exhibited an increase in nuclear area (Diamond et al., 1967; Diamond et al., 1975) while the hippocampal neurons did not show any change (Walsh and Cummins, 1979). It should be noted that in these studies, the rats were exposed to a novel enriched environment lasting for weeks before nuclear morphology was assessed. In contrast to the changes observed following long-term exposure to a novel environment, we found that the nucleus size either decreased or became less circular following short-term enrichment. However, consistent with previous findings made *in vitro*, we found that the percentage of neurons with an in-folded nuclear membrane increased following environmental enrichment (Wittmann et al., 2009).

In the current study, immunostaining for c-Fos protein was used to identify the somatosensory neurons and hippocampal CA1 place cells that were activated in response to novel environment exploration and the changes in nuclear dimensions were studied at 1 hour following exploration. The 1-h time was chosen because although cFos mRNA expression starts at 5 min and peaks by 30–45 min following neuronal stimulation, peak protein expression is seen around 60–90 min (Bullitt, 1990). It is known that external stimuli induce rapid activation of genes and changes in chromatin architecture that occur within minutes of neuronal stimulation (Sheng and Greenberg, 1990; Wittmann et al., 2009; Peter et al., 2021). However, the changes in nuclear dimensions that we observed at 1 h of exploration do not reveal the status of chromatin conformational and transcriptional changes. It will be interesting to study the dynamic changes of nuclear morphology in relation to activity-dependent transcription (for example, immediate-early gene expression) at different time points following neuronal activity. This could provide deeper insights into how synaptic activity influences nuclear architecture in relation of stimulus-dependent transcriptional changes.

We extended our study to evaluate how neuronal nuclear morphology may change under neurodegenerative disease conditions like Alzheimer’s disease (AD). We analyzed the neuronal nuclear morphology in neurons with increasing proximity to plaques in the APP/PS1 mouse model of AD. We found that the nuclear morphology in neocortical neurons in the immediate vicinity of the plaque remains unchanged for different plaque sizes. However, there was a significant reduction in the nuclear size and circularity at a radius of 40 µm from the plaque. This effect became more evident for bigger plaque sizes. A previous study using human autopsy brain sections found no change in nuclear diameter of CA4 pyramidal neurons and dentate granule neurons in the hippocampus but a decrease in nuclear diameter of oligodendrocytes (Gagyi et al., 2012). Another study also using human *post-mortem* samples of vascular dementia and AD patients found larger neuronal nuclei in specific populations of neurons in the hypothalamus of AD patients (Ishunina et al., 2019). Our findings suggest that the effective change in size and shape that the neuronal nuclei undergo in the immediate vicinity of Aβ plaques is independent of the plaque size. However, bigger plaques seem to have a much-pronounced influence on the nuclear morphology at increasing distances from them. In another interesting observation, it appears that there exists an invisible boundary (at around 60 µm from the plaque) beyond which the plaques, irrespective of their size, lose their influence over the neuronal nuclear morphology. All these observations could provide essential information about the progression of AD and add to the already existing knowledge of how nuclear dynamics is affected in AD (Iatrou et al., 2021). In fact, an earlier study using cancer cell lines had shown that shrinkage of nuclear size may be indicative of very late stages of apoptosis (Mandelkow et al., 2017). Therefore, the reduction in neuronal nuclear size may be reflective of an imminent cell death that occurs in AD. It will be interesting to see how nuclear morphology and dynamics would change from the prodromal stage of AD to symptomatic AD.

One limitation of this study is the use of epifluorescence microscope images (2-D images) to determine neuronal nuclear dimensions. With 2-D microscopy, measurements along a single plane introduces internuclear variability due to different placements and orientations of neuronal nuclei occupying different planes. Although the sections are closely bregma matched to ensure that similar regions are considered for analysis in different conditions, given the thickness of the sections (35–40 µm) used for immunostaining, it would be difficult to distinguish between nuclei that are intact *versus* those that were cut while sectioning. Therefore, it is possible that variations in quantifications are likely introduced due to neuronal nuclei present at different heights within the same brain section. One could predict the mean of the measurements from the frequency distributions. Frequency bar plots indicate the distributions of the number of nuclei for that specific parameter analysed (Supplementary Figures S1–S3). Frequency distributions also reflect the variability within the samples that may arise from the methodological constraints. The width and height of the curve indicate the stringency of measurements. A wider and relatively flatter curve indicates increased variation within the data whereas a narrower and taller curve signify lesser variations. Nevertheless, the above methodological inadequacies can be overcome using confocal microscopy followed by 3-D reconstruction of the nucleus and then considering only the intact nuclei for morphological analyses. This would reveal better insights into the finer details of nuclear architecture and the nature of the changes the nucleus undergoes in response to age, neuronal activity, and disease.

In summary, our study provides a collective view of the region-specific variations in neuronal nuclear dimensions in both young and aging animals. It also provides interesting insights about the changes in nuclear morphology under neurodegenerative disease conditions like Alzheimer’s disease. All these observations will likely lay the foundation for future studies to explore more about the nuclear changes that may serve as potential structural markers of aging and disease-associated deterioration of the mammalian brain.

## Data Availability

The original contributions presented in the study are included in the article/Supplementary Material, further inquiries can be directed to the corresponding author.
