# Supplementary material for: Region-specific heterogeneity in neuronal nuclear morphology in young, aged and in Alzheimer’s disease mouse brains
Source: Front Cell Dev Biol. 2023 Feb 1;11:1032504. doi: 10.3389/fcell.2023.1032504 (PMC9929567; doi:10.3389/fcell.2023.1032504)

Supplementary Figure Legends

Suppl. Figure 1. Frequency distributions of nuclear morphological features of neurons in neocortex and striatum. (A-B) Histograms showing frequency distribution for nuclear area, perimeter, and circularity at 3 months (top panels) and 24 months (bottom panels) of neocortical neurons (A) and striatal neurons (B).

Suppl. Figure 2. Frequency distributions of nuclear morphological features of neurons in hippocampus. (A-B) Histograms showing frequency distribution for nuclear area, perimeter, and circularity at 3 months (top panels) and 24 months (bottom panels) of hippocampal CA1 neurons (A) and CA3 neurons (B).

Suppl. Figure 3. Frequency distributions of nuclear morphological features of interneurons. (A) Histograms showing frequency distribution for nuclear area, perimeter, and circularity at 3 months for Calbindin-positive neurons in piriform cortex (PC) (top panels), somatosensory (SSC) and motor cortex (MC) (middle panels) and cerebellar Purkinje neurons (Cb) (bottom panels). **(B)** Histograms showing frequency distribution for nuclear area, perimeter, and circularity at 3 months for Parvalbumin-positive neurons in piriform cortex (PC) (top panels), somatosensory (SSC) and motor cortex (MC) (middle panels) and hippocampal CA1 region (bottom panels).

Suppl. Figure 1

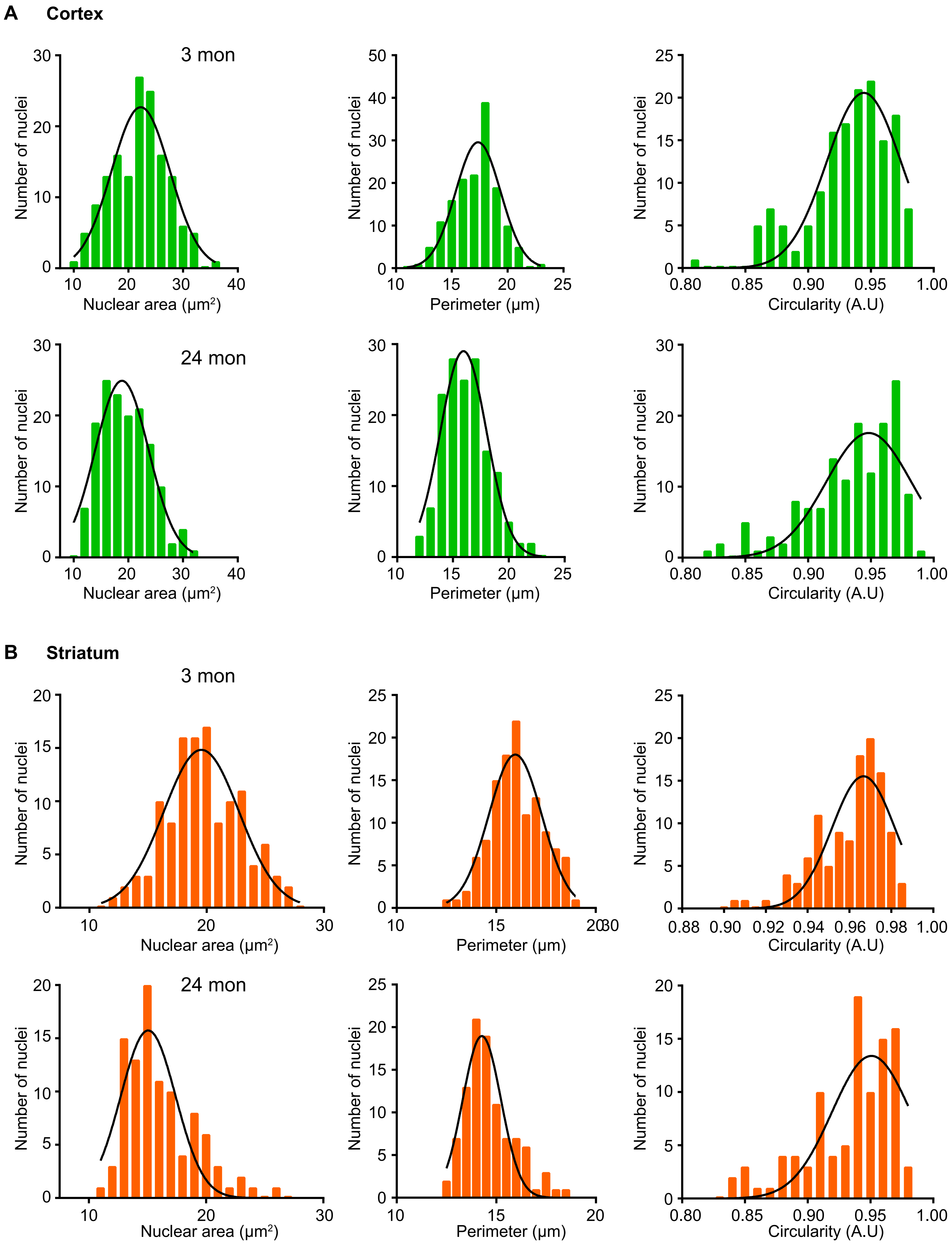

Suppl. Figure 2

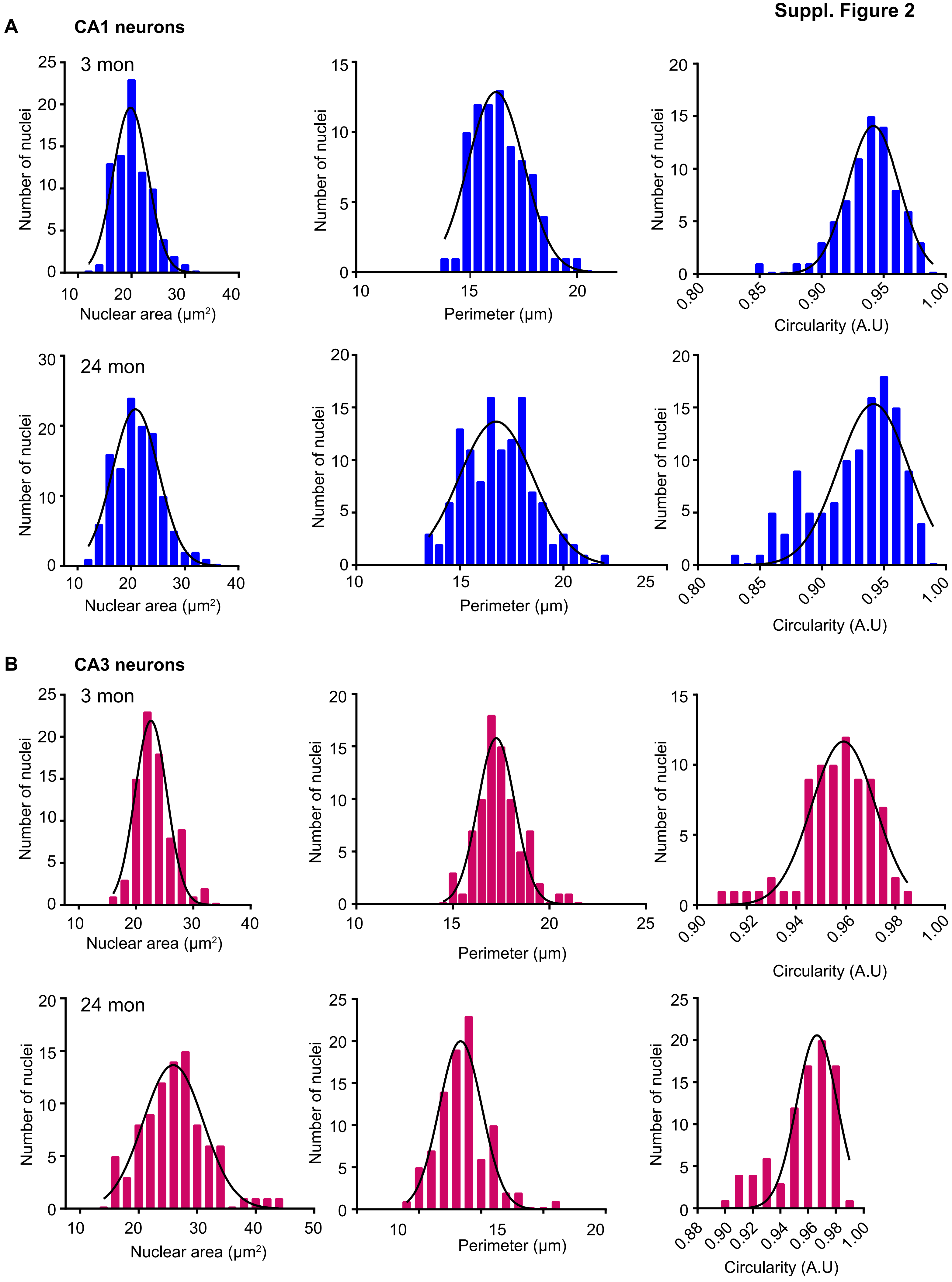

Suppl. Figure 3

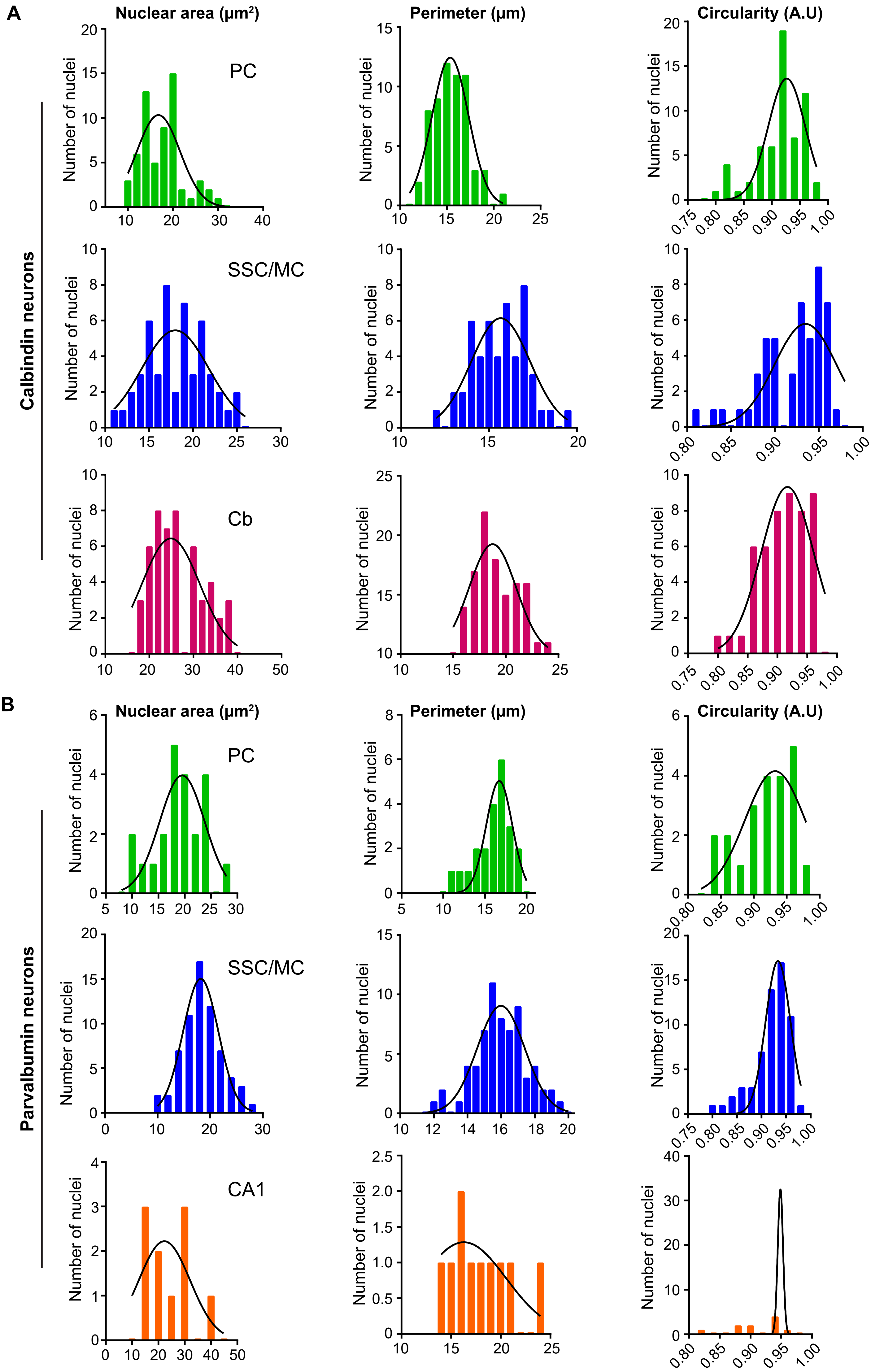

Supplement: Supplementary file 1 [file DataSheet1.PDF]
